# Airborne Warning System Direction-Finding Based on Real-Time SNR Correction

**DOI:** 10.3390/s26165253

**Published:** 2026-08-19

**Authors:** Jia Ding, Huaizong Shao, Haiwei Song, Jiawei Zhang, Fake Ding, Wen Zhang, Jie Liu, Jianxing Lv

**Affiliations:** 1 School of Information and Communication Engineering, University of Electronic Science and Technology of China, Chengdu 611731, China; dingji15@uestc.edu.cn; 2 Nanhu Laboratory, Defined RF Chip and System Research Center, Jiaxing 314001, China; hw8511@126.com (H.S.); jwnhlab@163.com (J.Z.);15757368626@163.com (F.D.); wzhang56799@163.com (W.Z.); liujie_scut@126.com (J.L.); 15852707697@163.com (J.L.); 3 School of Electronics and Information Engineering, Sichuan University, Chengdu 610065, China

**Keywords:** airborne warning system, direction-finding, attitude calibration, SNR correction, monopulse angle measurement, counter-UAV

## Abstract

The rapid advancement of unmanned aerial vehicle (UAV) technology has introduced increasingly severe airspace security challenges. As a critical component of counter-UAV systems, the direction-finding (DF) accuracy of airborne warning systems directly affects threat assessment and response efficiency. This paper addresses the problem of limited DF accuracy in airborne environments by proposing a direction-finding method that integrates attitude self-calibration with real-time signal-to-noise ratio (SNR) estimation. Based on the monopulse amplitude–phase comparison angle-measurement principle, the proposed method dynamically corrects the angle-discrimination curve using real-time SNR information and adaptively calibrates azimuth information by incorporating UAV attitude data. Simulation and experimental results demonstrate that the proposed method significantly reduces angle-measurement errors under low-SNR conditions, and attitude calibration further improves DF accuracy across the full angular range. Field experiments indicate that, after attitude calibration, the angle-measurement error is less than $2^{\circ }$ in over 77% of the test points.

## 1. Introduction

In recent years, the widespread adoption of UAV technology has generated extensive application scenarios in both civilian and military domains while simultaneously posing increasingly severe airspace security threats [1,2]. Incidents involving unauthorized UAV intrusions into critical infrastructure, airports, and sensitive areas occur frequently, presenting substantial challenges to public safety [3,4]. Consequently, the development of effective UAV detection and countermeasure technologies has become a research focus shared by academia and industry [5,6].

Radio frequency (RF) signal detection offers advantages including real-time responsiveness, wide coverage, and independence from optical conditions, and has been widely employed in UAV early warning systems [7,8]. RF detection systems intercept communication link signals between UAVs and their remote controllers to achieve target detection, identification, and localization [9]. Direction-finding (DF) technology constitutes the core function of RF detection, directly determining the system’s capability to ascertain the bearing of threat sources [10,11].

Conventional DF methods primarily include amplitude comparison and phase comparison techniques. Amplitude comparison methods estimate the direction of arrival by comparing amplitude differences across different antennas, but their accuracy degrades significantly under low-SNR conditions [12]. Phase comparison methods utilize phase differences between multiple antennas to measure angles with relatively high accuracy; however, they suffer from phase ambiguity issues and are sensitive to SNR variations [13]. Monopulse amplitude-phase comparison technology combines the advantages of both approaches by processing sum and difference channels to simultaneously exploit amplitude and phase information and has found mature applications in radar and electronic warfare domains [14,15].

Nevertheless, the application of monopulse DF technology to airborne platforms faces two prominent challenges. First, airborne environments typically exhibit elevated background noise and interference, causing significant fluctuations in received signal SNR. This leads to mismatches between the preset angle–discrimination curve and actual operating conditions, introducing systematic angle-measurement errors [16,17]. Second, attitude variations of the airborne platform (yaw, pitch, and roll) alter the relative relationship between the antenna coordinate system and the Earth coordinate system; if uncompensated, these variations directly cause DF result deviations [18]. For small UAV-borne platforms, where attitude perturbations are relatively pronounced, this issue is particularly significant [19].

To address these challenges, this paper proposes a direction-finding method for airborne warning systems comprising two core modules: real-time SNR correction and attitude adaptive calibration. The former estimates the received signal SNR online and dynamically adjusts the angle-discrimination curve to adapt to current signal quality; the latter utilizes attitude information provided by the onboard inertial measurement unit (IMU) to establish a transformation relationship from the antenna coordinate system to the ideal coordinate system, thereby eliminating the influence of platform attitude on DF results. The proposed method was validated on a UAV warning system prototype developed by Nanhu Laboratory, and field experimental results demonstrate that the method effectively improves DF accuracy in airborne environments [20].

This paper proposes an airborne direction-finding method for UAV warning systems that integrates real-time SNR adaptive correction and inertial attitude calibration. The core innovations are summarized as follows: existing SNR-adaptive monopulse methods in the current literature only perform parameter-level optimization, including SNR-based weight adjustment, error compensation and noise reduction gain tuning, while retaining a fixed offline-calibrated angle-discrimination curve. Such schemes fail to adapt to dynamic SNR fluctuations in complex electromagnetic scenarios. Different from these conventional methods, the proposed scheme realizes curve-level SNR adaptation, which directly generates the optimal angle-discrimination curve corresponding to the real-time signal quality. Furthermore, unlike traditional independent attitude-compensation methods that only focus on platform stabilization, a multi-stage coordinate transformation from the antenna coordinate system to the ideal horizontal coordinate system is deeply embedded into the entire monopulse direction-finding workflow, which effectively eliminates the direction-finding errors caused by UAV attitude deflection.

## 2. Airborne Warning System Overview

Based on multi-channel UAV payload prototype key technologies, the Reconfigurable RF Chip and System Center at Nanhu Laboratory developed a UAV airborne warning system prototype. This device does not rely on decoding target UAV broadcast information; instead, it directly detects and intercepts signal amplitude-phase characteristics to achieve target bearing tracking and warning.

### 2.1. System Design Specifications

The fundamental design specifications of the warning system are summarized in Table 1. As shown in Table 1, the system supports multi-band signal monitoring within the 800 MHz∼6 GHz range and can identify mainstream UAV brands including DJI and Autel. The system features a single-channel instantaneous bandwidth exceeding 150 MHz, with a minimum detectable signal level below −105 dBm @ 1 MHz bandwidth. It supports simultaneous detection of no fewer than five targets, with an effective detection range exceeding 800 m. The equivalent isotropic radiated power exceeds 2 W, and the detection response time is less than 2 s. The device volume is less than 465 mm × 135 mm × 50 mm, with a weight below 1 kg (excluding battery) and total power consumption under 40 W.

### 2.2. Hardware Architecture

**Figure 1:**
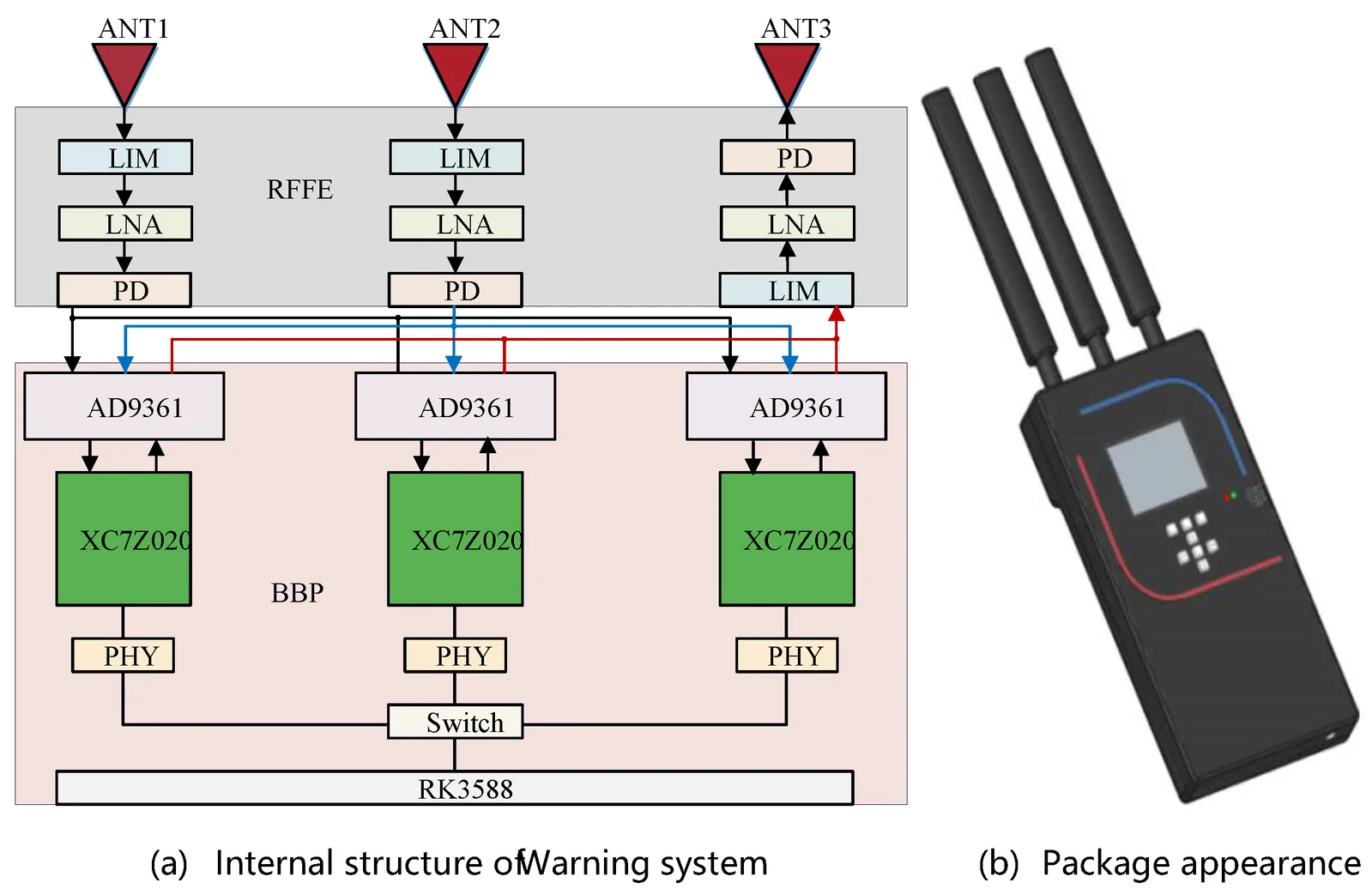
Basic structure of the wideband phased-array RF system: (**a**) schematic diagram of the system composition; (**b**) physical prototype of the warning system.

The handheld UAV warning system consists primarily of antennas, an RF-digital integrated module, a power supply module, and structural components. The system architecture is illustrated in Figure 1.

As shown in Figure 1a, the antenna subsystem includes two receiving antennas and one transmitting antenna for electromagnetic wave-to-electrical signal conversion. The RF front-end performs RF reception preprocessing and transmission power amplification, supporting two receive channels and one transmit channel. One receive preprocessing path converts RF signals through limiting, attenuation, and low-noise amplification (LNA), then splits the signal via a power divider to three AD9361 R1A ports; the other receive preprocessing path outputs them to the AD9361 R2A port. During transmission, the RF front-end combines three AD9361 T1A output signals, amplifies them through a power amplifier, and outputs to the transmit antenna. The baseband processing module completes AD9361 driving, power spectrum estimation, multi-channel data synthesis, intelligent analysis and identification, and interference excitation generation, enabling UAV detection, warning, and low-power self-defense jamming.

Figure 1b presents the physical prototype of the warning system. Except for the antennas, all components are integrated within a custom enclosure. The basic configuration list is provided in Table 2.

The warning system comprises three core components: antennas (ANT), RF front-end module (RFFE), and baseband processing module (BBP), along with other accessories.

## 3. Angle-Measurement Principles

Amplitude comparison and phase comparison are common monopulse angle-measurement methods widely employed in various direction-finding devices. However, SNR variations can increase direction-finding errors, and phase comparison methods are prone to ambiguous solutions. Airborne platforms are typically small UAVs, and direction-finding results are highly susceptible to platform attitude effects; even for large aircraft, the influence of aircraft attitude on target measurements cannot be ignored. Therefore, this paper proposes an airborne warning system direction-finding method based on attitude self-calibration and real-time SNR estimation to further improve direction-finding accuracy.

**Figure 2:**
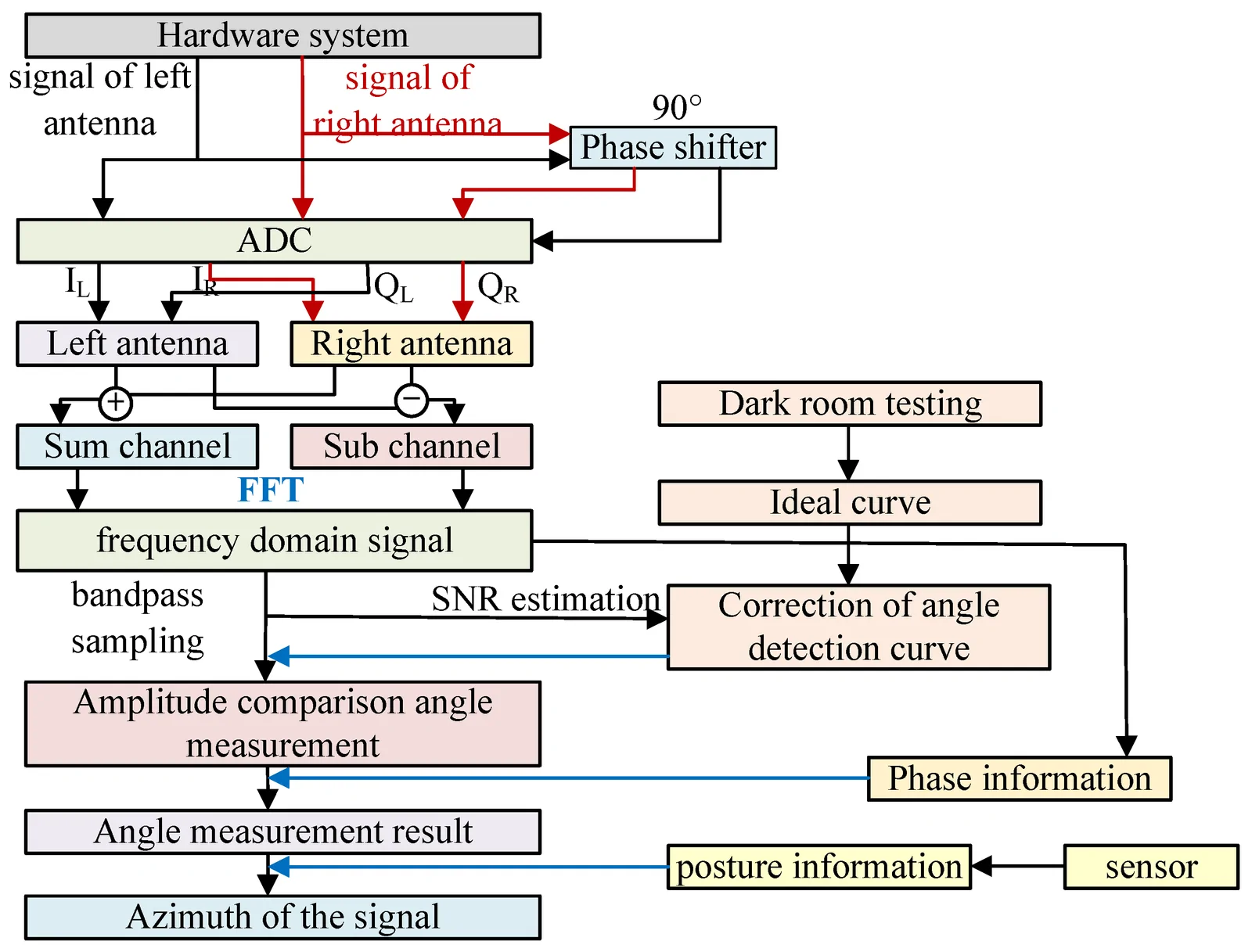
Direction-finding workflow of the airborne warning system.

As shown in Figure 2, signals from the target UAV are received by the warning system’s antennas and, after amplification and filtering by the hardware system, are converted to digital signals by the analog-to-digital converter (ADC). The data processing system combines the acquired I/Q orthogonal signals into complex signals and transforms them to the frequency domain via Fourier transform. In the frequency domain, the peak region of the target sum-channel signal is identified, and both the signal power (including noise) and average noise power are calculated.

The SNR information is passed to the angle-discrimination curve calibration function to obtain the corresponding discrimination curve under the current SNR condition. Combined with the corrected angle-discrimination curve and sum-difference channel signals, monopulse amplitude comparison angle measurement is completed. Since the angle-discrimination curve is symmetric about the boresight direction of the two antennas, phase information is further utilized to resolve ambiguity. Finally, the UAV attitude information is incorporated to complete the final correction of target azimuth information.

### 3.1. Fundamental Angle-Measurement Principles

#### 3.1.1. Phase Comparison Angle Measurement

Phase comparison angle measurement is commonly used with phased-array antennas and, combined with multi-baseline angle-measurement methods, can achieve target localization. This method requires at least two independent antennas with identical physical orientations, capable of measuring one-dimensional azimuth information. With only one baseline, to avoid ambiguous solutions, the spacing between the two antennas must be less than half a wavelength.

**Figure 3:**
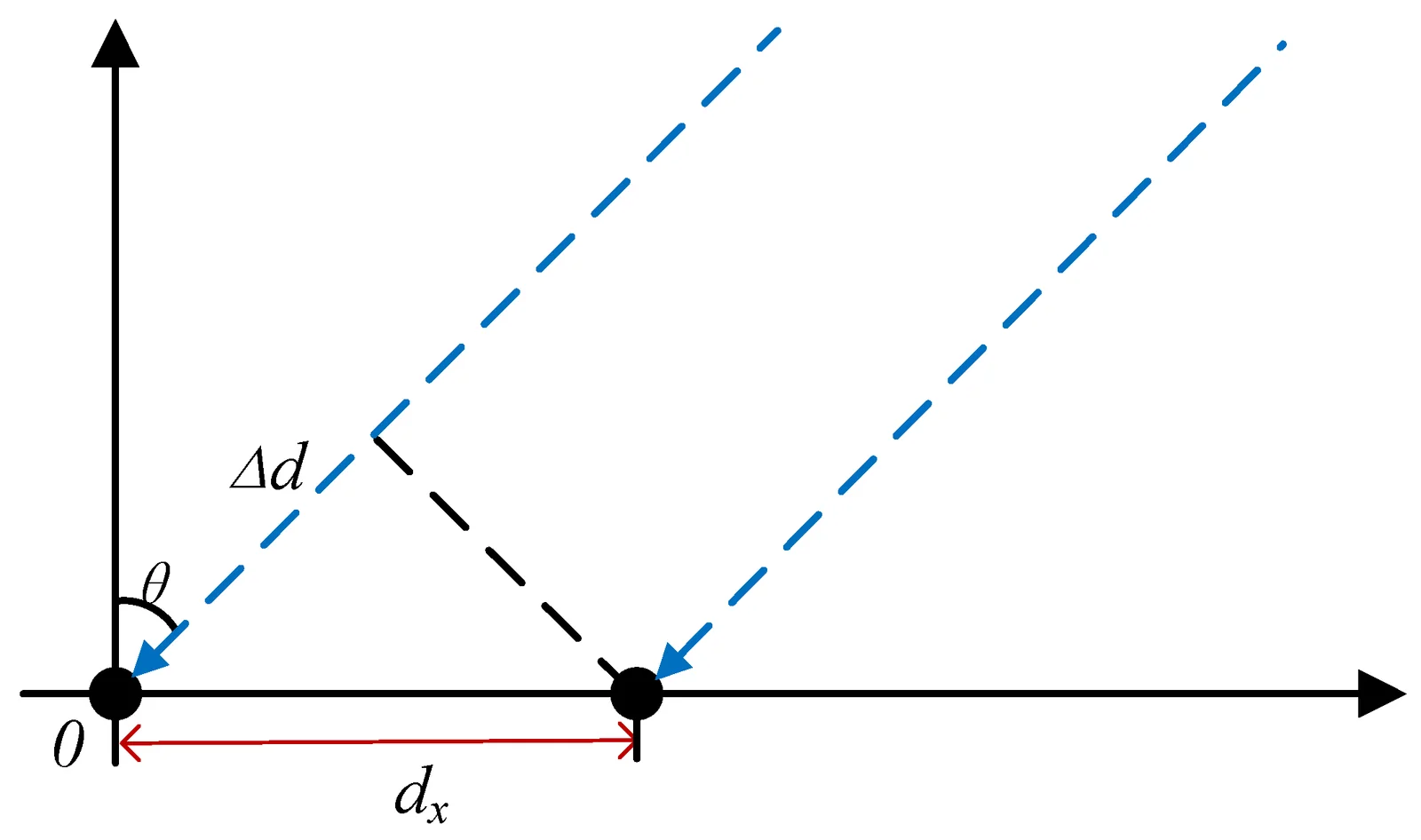
One-dimensional antenna configuration.

As shown in Figure 3, for two parallel antennas, the signal path difference when arriving at different antenna positions is:

(1) $$\Delta d=d_{x}\sin\theta ,$$

The phase difference between adjacent antenna channels is:

(2) $$\Delta \Phi =\frac{\Delta d}{\lambda }2\pi ,$$

When the phase information between adjacent antenna channels is extracted from the I/Q data, the path difference Δd=ΔΦλ/2π is first calculated according to Equation (2), and then the angle $\theta =\arcsin(\Delta d/d_{x})$ relative to the antenna boresight direction is obtained according to Equation (1).

#### 3.1.2. Amplitude–Phase Comparison Angle Measurement

**Figure 4:**
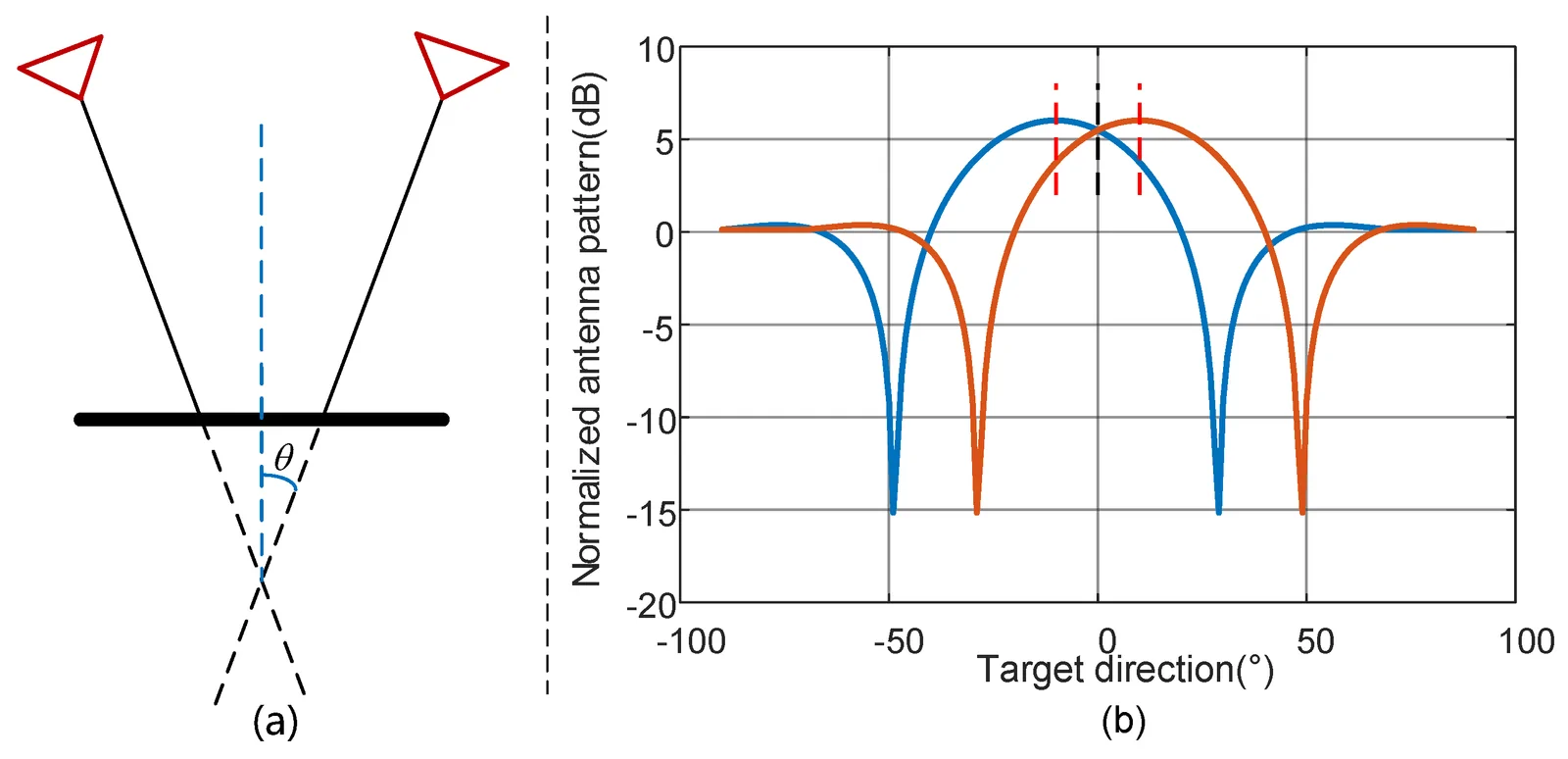
Antenna placement and radiation pattern: (**a**) antenna placement schematic; (**b**) antenna pattern at 6 GHz.

In contrast to the configuration in Section 3.1.1, the antennas are placed in a V-shaped arrangement, with a relative angle of $30^{\circ }$ between the two antennas. Figure 4 shows the antenna placement schematic and radiation patterns.

As shown in Figure 4a, the two antennas are positioned at $\pm 10^{\circ }$ relative to the boresight, with an equivalent spacing of 6 cm. Figure 4b presents the received antenna power variation with target azimuth at 6 GHz. During field testing, the measured data are substituted into the pre-calibrated angle-discrimination curve to solve for the target direction. Based on the sum and difference channel information of the two antennas, the angle-discrimination curve is generated. Let the radiation patterns of the left and right antennas be $F_{nL}$ and $F_{nR}$, respectively; the sum-difference channel outputs and angle-discrimination curve are:

(3) $$\Delta =F_{nL}-F_{nR},$$

(4) $$\Sigma =\left|F_{nL}+F_{nR}\right|,$$

(5) $$f\left(\theta \right)=\frac{\Delta }{\Sigma },$$

**Figure 5:**
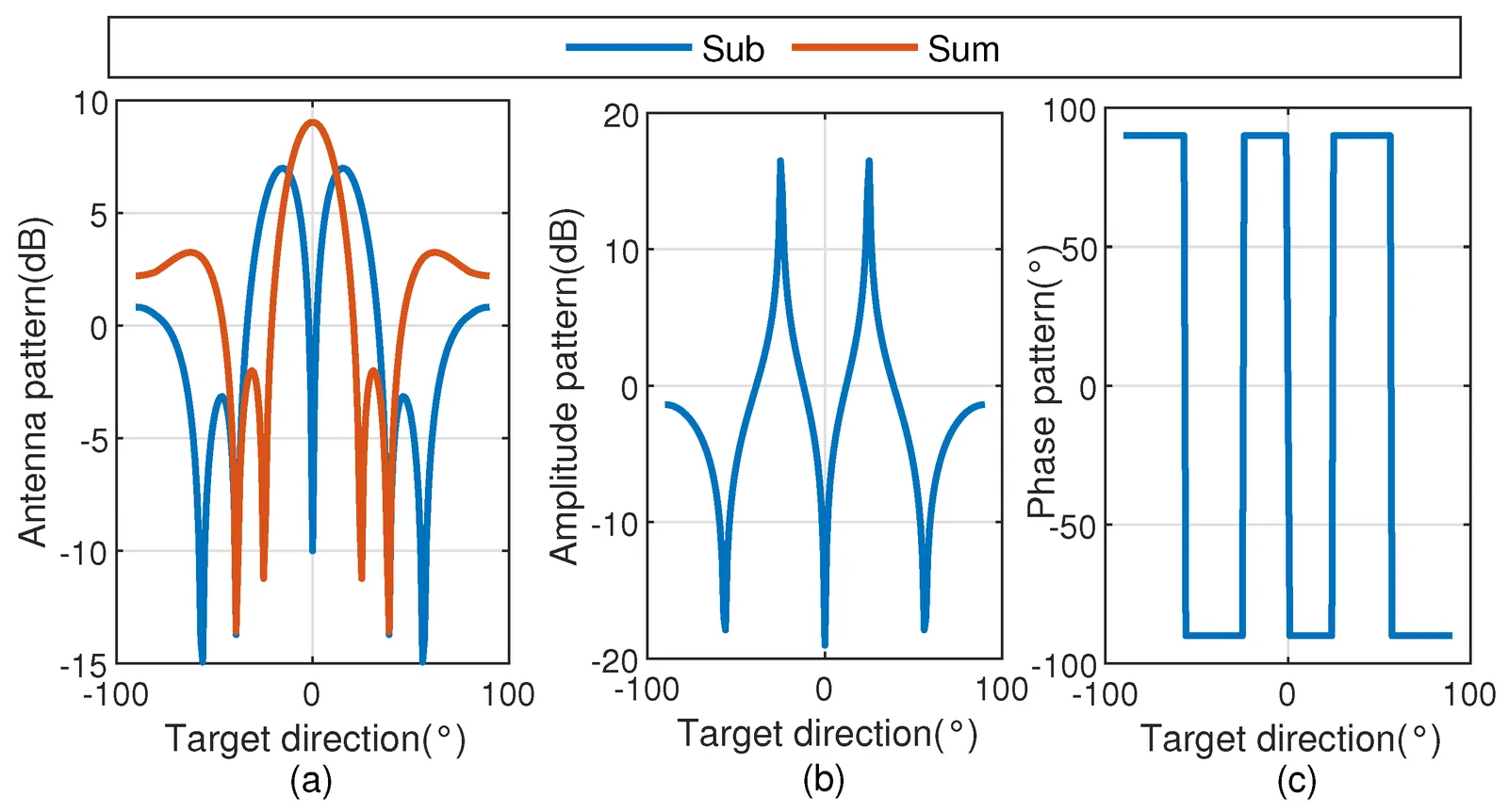
Angle-discrimination curves: (**a**) sum and difference channel antenna patterns; (**b**) angle-discrimination curve; (**c**) phase curve.

Substituting the simulation data from Figure 4b into Equation (5), the sum and difference channel antenna patterns, amplitude angle-discrimination curve, and phase curve are obtained, as shown in Figure 5a.

In Figure 5b, the angle-discrimination curve is monotonic within the $\left[-25^{\circ },0^{\circ }\right]$ and $\left[0^{\circ },25^{\circ }\right]$ regions. Based on the measured sum-difference ratio, the target direction can be calculated. However, the angle-discrimination curve is symmetric about $0^{\circ }$, which produces ambiguous solutions during target azimuth estimation. In this case, the phase curve shown in Figure 5c is utilized for judgment to achieve positive/negative direction discrimination of the target azimuth.

### 3.2. Real-Time Angle-Discrimination Curve Correction

When noise is considered in the above equations, let $S_{L}$, $S_{R}$ represent the signals and $N_{L}$, $N_{R}$ represent the noise; Equation (5) can be rewritten as:

(6) $$f_{noisy}\left(\theta \right)=\frac{|\left(S_{L}+N_{L}\right)-\left(S_{R}+N_{R}\right)|}{\left(S_{L}+N_{L}\right)+\left(S_{R}+N_{R}\right)},$$

In Equation (6), let $S_{\Sigma }=S_{L}+S_{R}$ and $S_{\Delta }=S_{L}-S_{R}$; substituting these into Equation (6) yields:

(7) $$f_{noisy}\left(\theta \right)=\frac{S_{\Delta }+\left|N_{L}-N_{R}\right|}{S_{\Sigma }+\left|N_{L}+N_{R}\right|},$$

In Equation (7), since noise and signals are uncorrelated, the absolute value operators in the numerator and denominator can be further decomposed. The noise in the left and right antennas is also uncorrelated, so $|N_{L}-N_{R}\left|\;\approx \;\right|N_{L}+N_{R}|$. Focusing on the sum-channel signal $S_{\Sigma }$ and noise $N_{\Sigma }$, the sum-channel can be expressed as $SNR_{\Sigma }=|S_{L}+S_{R}\left|/\right|N_{L}+N_{R}|$; the angle-discrimination curve under ideal conditions is $f_{ideal}\left(\theta \right)=S_{\Delta }/S_{\Sigma }$. Substituting this into Equation (7) and rewriting gives:

(8) $$\begin{array}{cc} f_{noisy}\left(\theta \right) & =\frac{f_{ideal}\left(\theta \right)\cdot \left|S_{L}\left(\theta \right)+S_{R}\left(\theta \right)\right|+\frac{|S_{L}\left(\theta \right)+S_{R}\left(\theta \right)|}{SNR_{\Sigma }}}{|S_{L}\left(\theta \right)+S_{R}\left(\theta \right)|+\frac{|S_{L}\left(\theta \right)+S_{R}\left(\theta \right)|}{SNR_{\Sigma }}}\\ \; & =\frac{f_{ideal}\left(\theta \right)+\frac{1}{SNR_{\Sigma }}}{1+\frac{1}{SNR_{\Sigma }}}\\ \; & =\frac{f_{ideal}\left(\theta \right)\cdot SNR_{\Sigma }+1}{SNR_{\Sigma }+1} \end{array}$$

Anechoic chamber testing can obtain the angle-discrimination curve $f_{measured}\left(\theta \right)$ under high-SNR conditions, with the corresponding SNR denoted as $SNR_{ref}$. Based on this SNR and the measured discrimination curve, the ideal angle-discrimination curve can be solved:

(9) $$f_{ideal}\left(\theta \right)=\frac{f_{measured}\left(\theta \right)\cdot \left(SNR_{ref}+1\right)-1}{SNR_{ref}},$$

Once $f_{ideal}\left(\theta \right)$ is obtained, it can be substituted into Equation (8) to generate the angle-discrimination curve under arbitrary SNR conditions.

### 3.3. Attitude Adaptive Calibration

**Figure 6:**
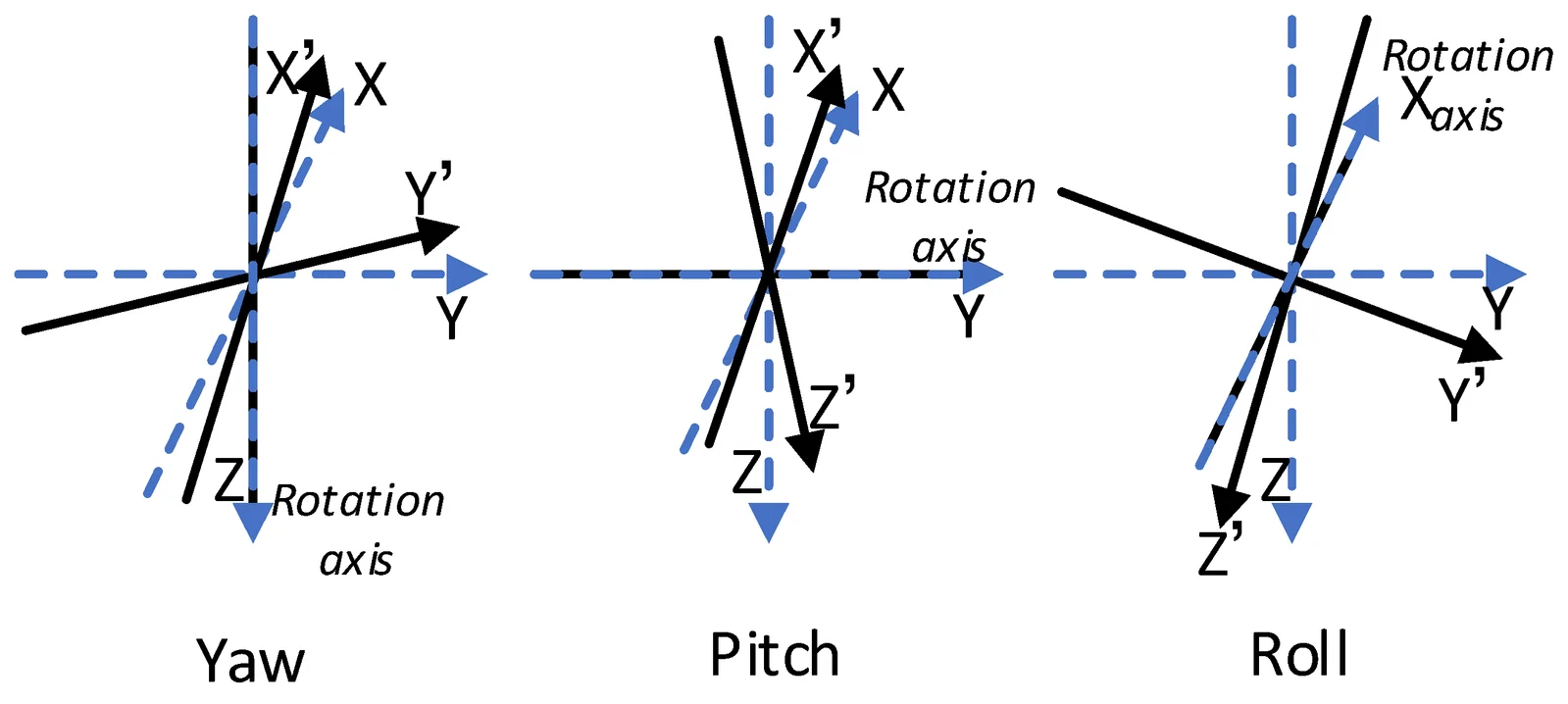
Attitude coordinate system.

To analyze the influence of UAV attitude on direction-finding accuracy, an attitude coordinate system is established with the UAV’s ideal position as the origin, as shown in Figure 6.

As shown in Figure 6, the positive directions of the three attitude rotation angles are defined as follows:

In Table 3, the definition sequence of the three attitude deflections is arranged as yaw, pitch and roll in turn. The representation of a unit vector in the original coordinate system after UAV attitude rotation in the new coordinate system is:

(10) $$\begin{array}{c} \left[\begin{array}{c} R_{x}\\ R_{y}\\ R_{z} \end{array}\right]=\left[\begin{array}{ccc} 1 & 0 & 0\\ 0 & \cos\theta_{roll} & \sin\theta_{roll}\\ 0 & -\sin\theta_{roll} & \cos\theta_{roll} \end{array}\right]\;\left[\begin{array}{ccc} \cos\theta_{pitch} & 0 & -\sin\theta_{pitch}\\ 0 & 1 & 0\\ \sin\theta_{pitch} & 0 & \cos\theta_{pitch} \end{array}\right]\;\left[\begin{array}{ccc} \cos\theta_{yaw} & \sin\theta_{yaw} & 0\\ -\sin\theta_{yaw} & \cos\theta_{yaw} & 0\\ 0 & 0 & 1 \end{array}\right]\;\left[\begin{array}{c} O_{x}\\ O_{y}\\ O_{z} \end{array}\right]\\ \;\;R_{xyz}=M_{\theta_{roll}}\cdot M_{\theta_{pitch}}\cdot M_{\theta_{yaw}}\cdot O_{xyz} \end{array}$$

**Figure 7:**
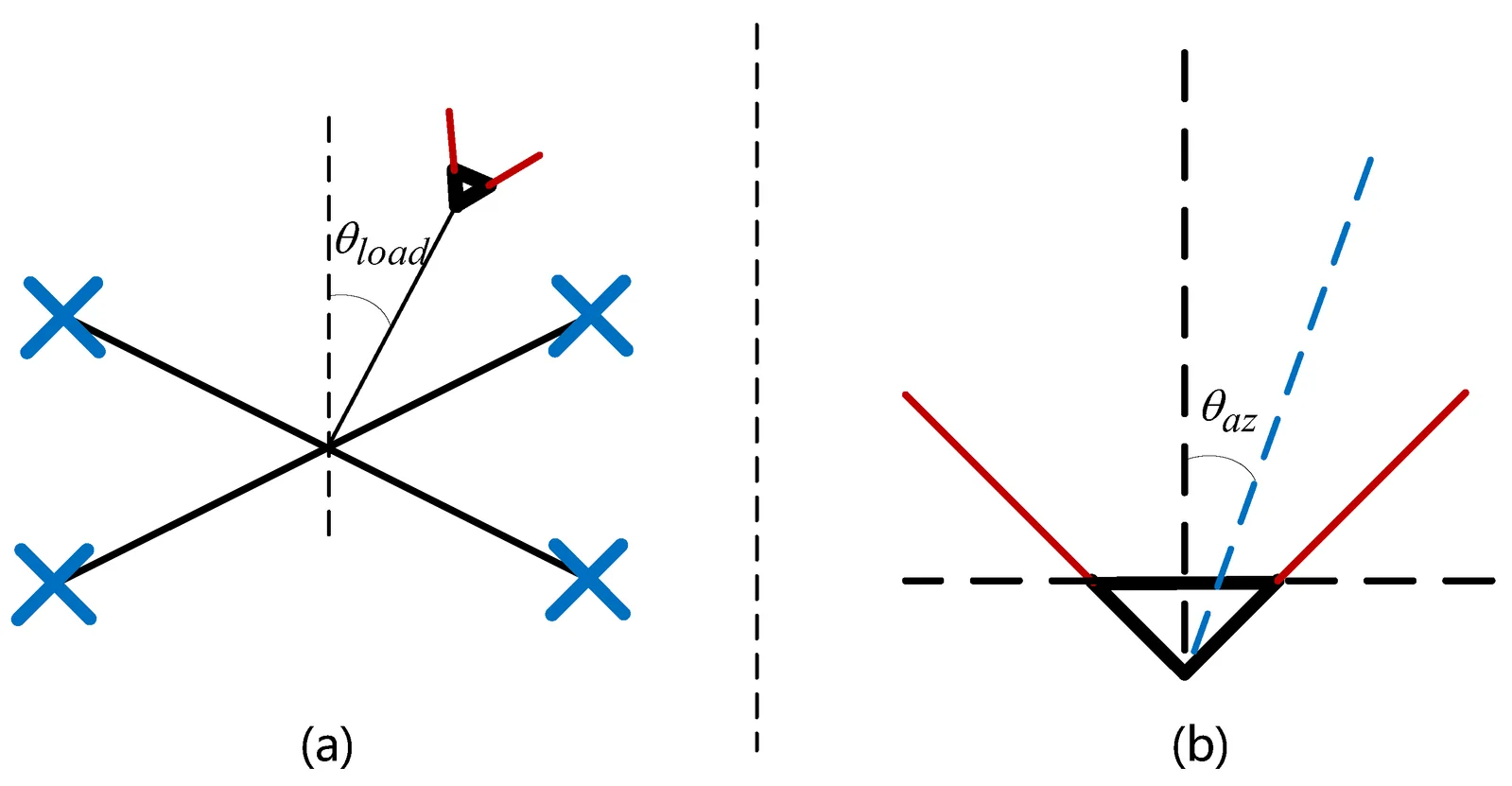
Relative position: (**a**) Schematic diagram of load position; (**b**) Target in the antenna coordinate system.

In Equation (10), $O_{xyz}$ and $R_{xyz}$ denote the original fixed coordinate system (with the positive *X*-axis pointing north and the *Z*-axis toward the Earth’s center) and the body-fixed coordinate system established with respect to the UAV, respectively. Furthermore, in coordinate system $R_{xyz}$, considering that the actual installation orientation of the payload is $\theta_{load}$ (mounted within the *X*-*Y* plane of coordinate system $R_{xyz}$), the schematic diagram and the corresponding equations for the payload installation are shown in Figure 7a and Equation (11), respectively.

(11) $$\begin{array}{cc} \; & \left[\begin{array}{c} A_{x}\\ A_{y}\\ A_{z} \end{array}\right]=\left[\begin{array}{ccc} \cos\theta_{load} & \sin\theta_{load} & 0\\ -\sin\theta_{load} & \cos\theta_{load} & 0\\ 0 & 0 & 1 \end{array}\right]\;\left[\begin{array}{c} R_{x}\\ R_{y}\\ R_{z} \end{array}\right]\\ \; & A_{xyz}=M_{\theta_{load}}\cdot R_{xyz} \end{array}$$

As shown in Figure 7b, in the antenna coordinate system $A_{xyz}$, when the target lies in the antenna plane, the target azimuth vector can be expressed as $\left[A_{x},A_{y},A_{z}\right]=\left[\cos\left(\theta_{az}\right),\sin\left(\theta_{az}\right),0\right]$. Transforming this vector to the ideal coordinate system yields:

(12) $$O_{xyz,aim}=M_{\theta_{yaw}}^{-1}\cdot M_{\theta_{pitch}}^{-1}\cdot M_{\theta_{roll}}^{-1}\cdot M_{\theta_{load}}^{-1}\cdot A_{xyz,aim},$$

## 4. Simulation Analysis

Referencing the parameters of the warning system under development at Nanhu Laboratory, simulation analysis was conducted on the correlation between SNR and angle-discrimination curve effects. Gaussian white noise was introduced in the simulation to model the influence of different SNRs on angle-measurement accuracy. Simulated sum-difference channel data were substituted into the angle-discrimination curve to statistically evaluate angle-measurement accuracy. The angle-discrimination curves included the ideal curve and two types of SNR-introduced curves, totaling three categories. The simulation configuration parameters are listed in Table 4.

### 4.1. Angle-Measurement Errors Induced by SNR

**Figure 8:**
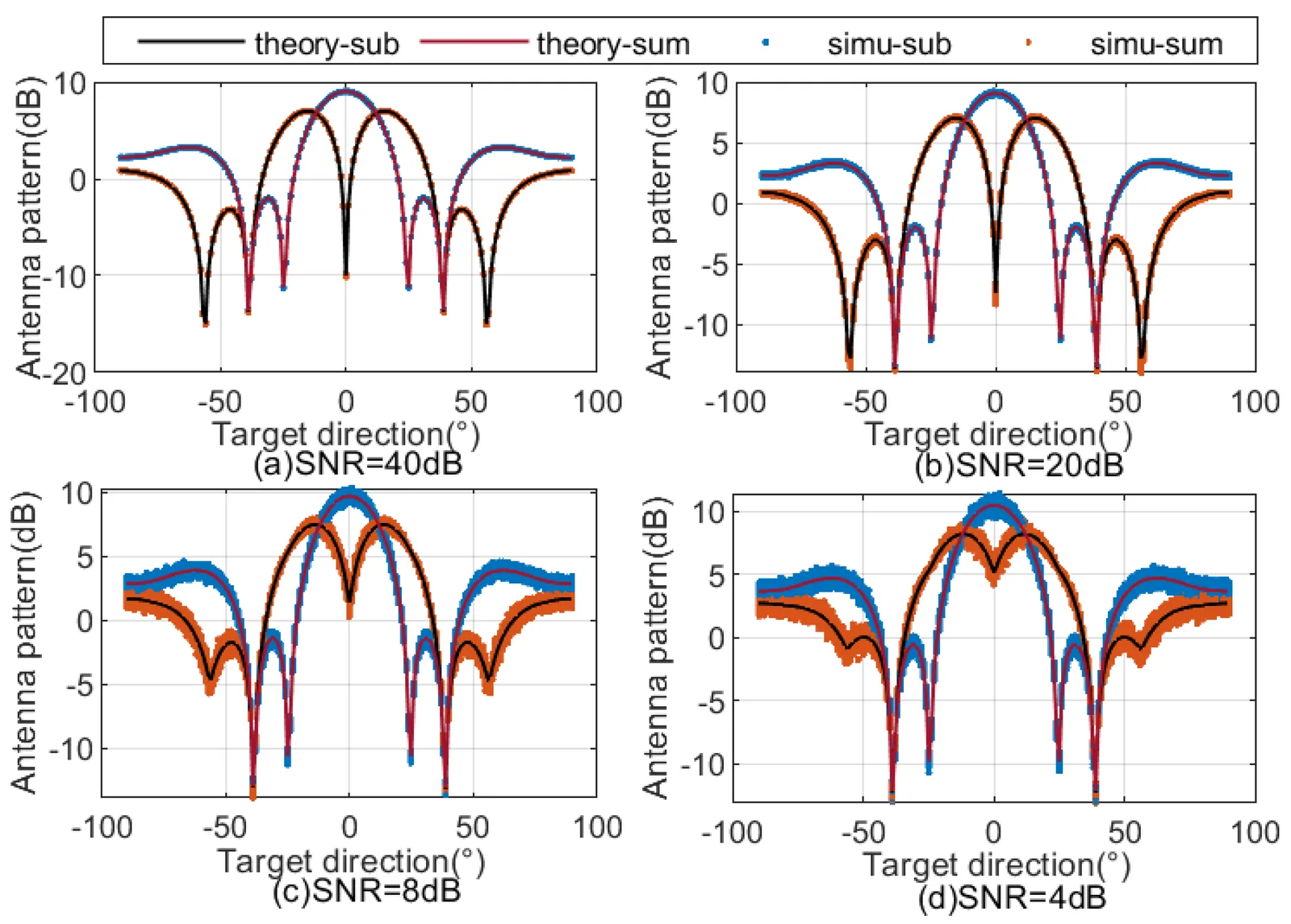
Sum and difference channels under low-SNR conditions: (**a**) 40 dB; (**b**) 20 dB; (**c**) 8 dB; (**d**) 4 dB.

The antenna radiation patterns were obtained through HFSS simulation, and their parameters were introduced into the simulation program. Figure 8 presents the sum and difference channel images corresponding to sum-channel SNRs of 40 dB, 20 dB, 8 dB, and 4 dB.

Figure 8 shows the simulation results under low-SNR conditions. With a fixed sum-channel SNR, the image data for different wave arrival angles are plotted. Subfigures (a), (b), (c), and (d) correspond to SNRs of 40 dB, 20 dB, 8 dB, and 4 dB, respectively. A total of 181 target points in the $-90^{\circ }$ to $90^{\circ }$ range were simulated, with 100 Monte Carlo random trials conducted for each point. Blue scatter points represent the distribution of sum-channel data with target angle; orange scatter points represent difference-channel data with target angle; red and black solid lines represent the theoretical values of sum and difference channels under corresponding conditions, respectively.

From Figure 8, it can be observed that as the SNR gradually decreases, the dispersion of sum-difference channels gradually increases. For high-SNR conditions, whether 40 dB or 20 dB, the impact on curve shape is relatively small. As the SNR decreases, this impact becomes increasingly pronounced. A low SNR primarily affects regions with weak signals, and the valley of the ideal difference-channel curve gradually rises.

**Figure 9:**
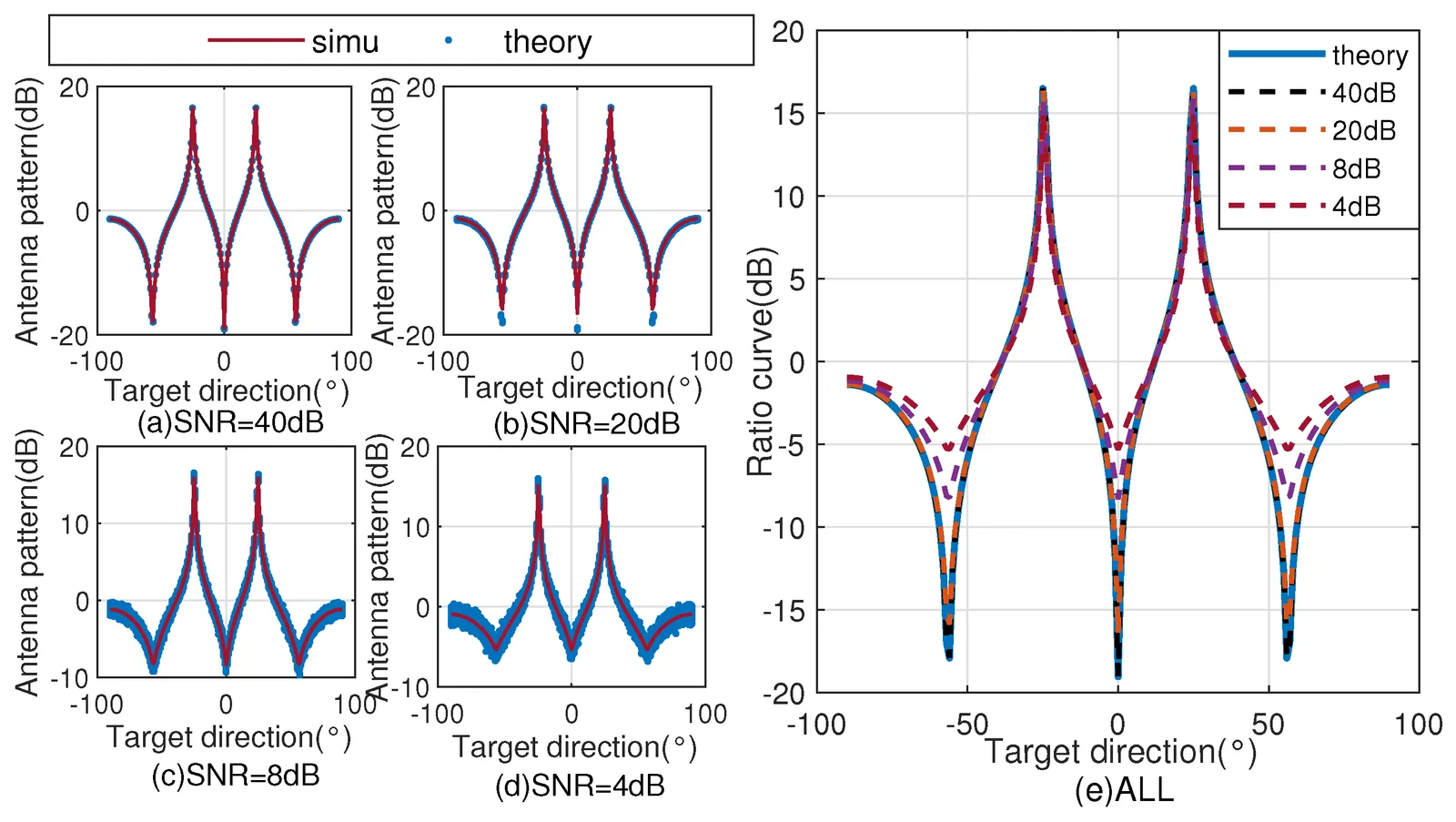
Angle-discrimination curves under low-SNR conditions: (**a**) 40 dB; (**b**) 20 dB; (**c**) 8 dB; (**d**) 4 dB; (**e**) comparison of theoretical angle-discrimination curves under different SNRs.

Dividing the sum and difference channel data from Figure 8 yields the corresponding angle-discrimination curves shown in Figure 9.

Figure 9 presents the sum-difference channel ratio results, where (a), (b), (c), and (d) correspond to SNRs of 40 dB, 20 dB, 8 dB, and 4 dB, respectively. Blue scatter points represent the distribution of ratio results with target angle; red solid lines represent the theoretical angle-discrimination curves under corresponding SNR conditions. It can be observed that as the SNR decreases, the dispersion of target signal ratio results gradually increases.

Figure 9e compares the theoretical angle-discrimination curves under different SNR conditions. The blue solid line represents the ideal condition (no noise and infinite SNR); the orange, yellow, purple, and green dashed lines represent the theoretical angle-discrimination curves at 40 dB, 20 dB, 8 dB, and 4 dB, respectively. It can be seen that under large-SNR conditions, the angle-discrimination curve is essentially consistent with the ideal curve; as the SNR decreases, the valley at $0^{\circ }$ gradually rises, and the overall slope of the angle-discrimination curve gradually decreases.

**Figure 10:**
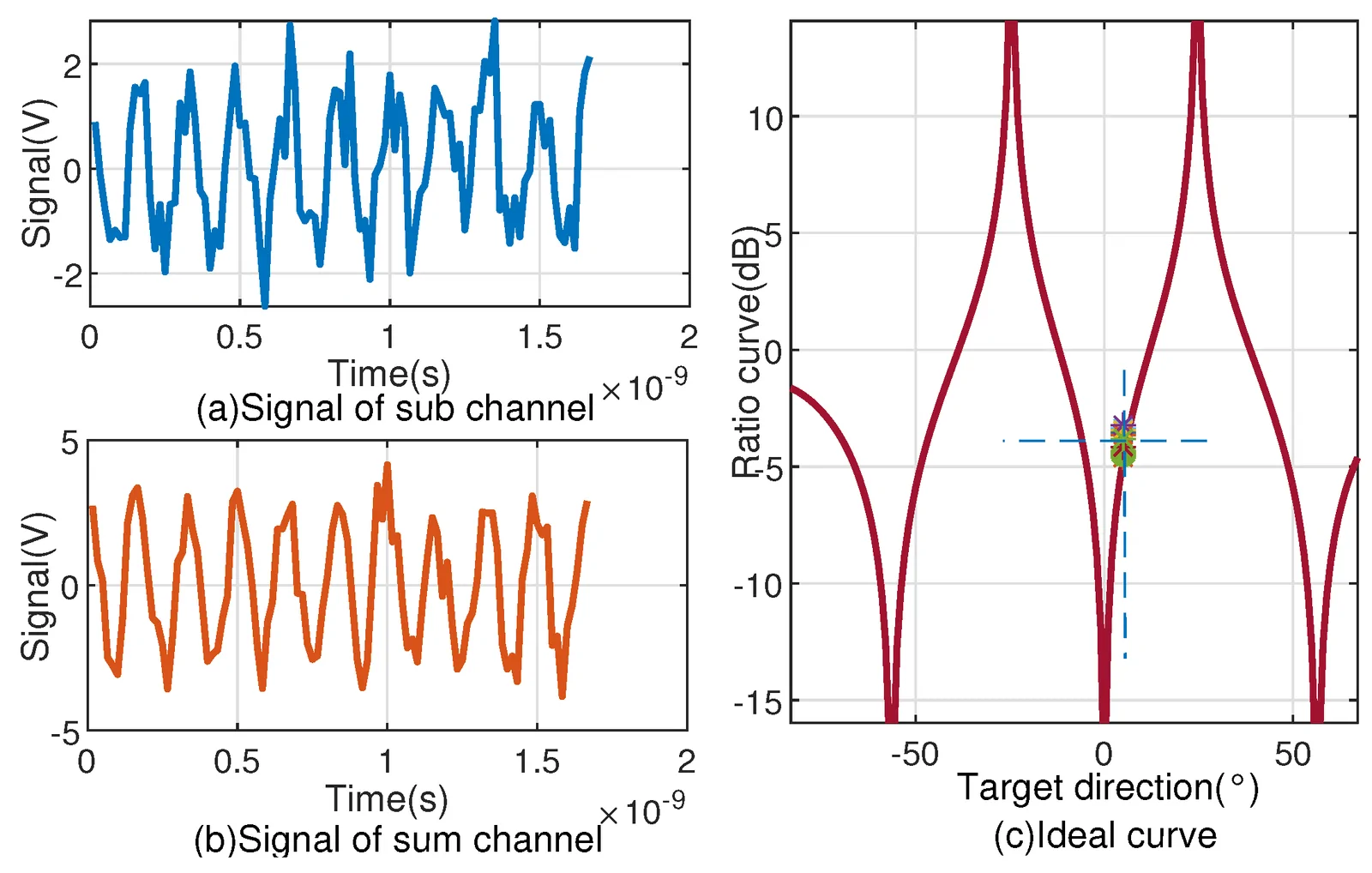
Sum-difference signals and angle-measurement results: (**a**) sum-channel signal; (**b**) difference-channel signal; (**c**) ideal angle-discrimination curve and angle-measurement results.

To investigate the influence of a low SNR on angle-measurement results, single-point test results were substituted into the corresponding angle-discrimination curves to obtain the angle-measurement results. Figure 10 shows the sum-difference channel signal images and corresponding angle-measurement results when the snapshot number is 100.

In Figure 10, (a) and (b) represent the sum and difference channel signals, respectively, corresponding to a target azimuth of $5^{\circ }$ and SNR of 8 dB, with a simulation sampling frequency of 60 GHz. A total of 100 data points over 1.67 ns were extracted, and the average power was calculated to obtain the ratio result.

Figure 10c shows the ideal angle-discrimination curve. Substituting the ratio results from 10 simulations, all points lie above the ideal angle-discrimination curve. One point has the coordinates ($5^{\circ }$, −3.81 dB); when solving, the Y-coordinate value is substituted into the angle-discrimination curve to re-solve for the X-coordinate, yielding approximately $6^{\circ }$, with an angle-measurement error of about $1^{\circ }$.

**Figure 11:**
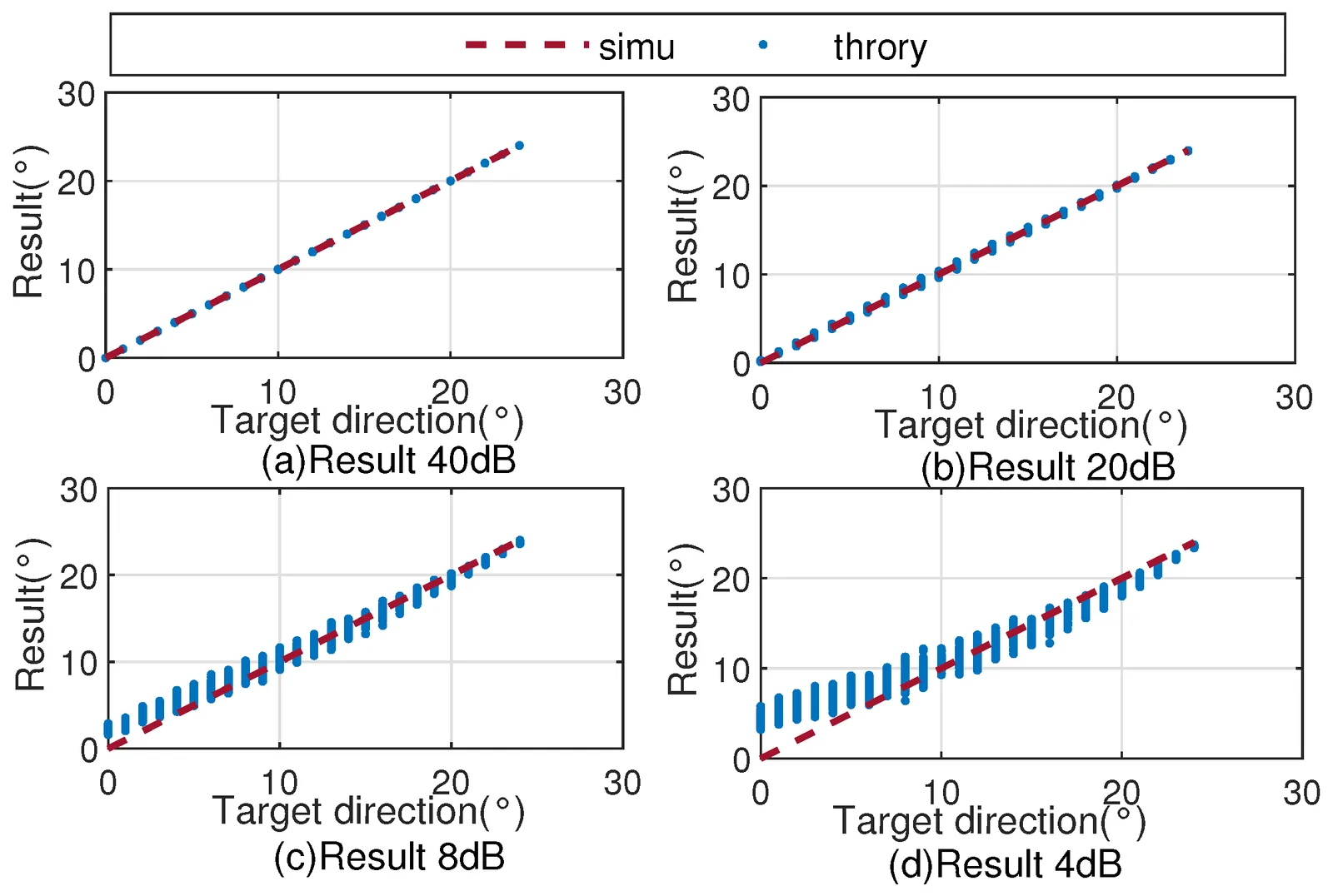
Angle-measurement results: (**a**) 40 dB; (**b**) 20 dB; (**c**) 8 dB; (**d**) 4 dB.

Extending the simulation angle to 25 azimuth points from $0^{\circ }$ to $24^{\circ }$ and conducting 100 repeated simulations, the results are shown in Figure 11.

**Figure 12:**
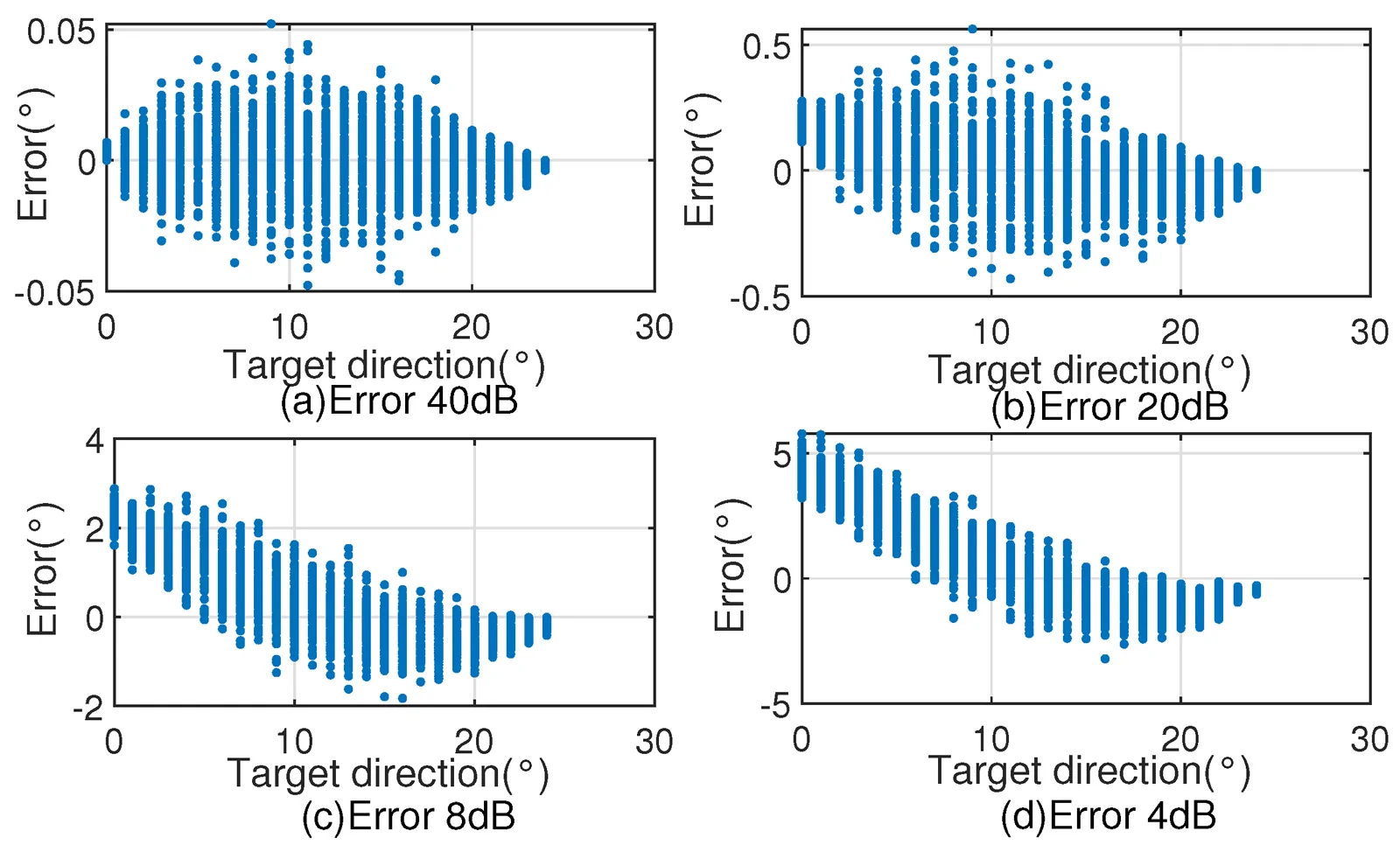
Angle-measurement errors: (**a**) 40 dB; (**b**) 20 dB; (**c**) 8 dB; (**d**) 4 dB.

Figure 11 presents the angle-measurement results, where (a), (b), (c), and (d) correspond to SNRs of 40 dB, 20 dB, 8 dB, and 4 dB, respectively. Blue scatter points represent the results solved using the ideal angle-discrimination curve; red dashed lines represent the true values. It can be observed that as the SNR decreases, the deviation between angle-measurement results and theoretical values gradually increases. Subtracting the solved results from the actual values yields the error images shown in Figure 12.

From the angle-measurement error images shown in Figure 12, it can be seen that as the SNR decreases, angle-measurement errors gradually increase. Under the same SNR condition, the errors are largest near $0^{\circ }$ and at large angles.

Table 5 presents the statistical angle-measurement errors under different target angles and SNR conditions.

In practical applications, when the target wave arrival angle is limited within the effective angle-discrimination range, angle-measurement errors are significant under poor SNR conditions and small target arrival angles.

Therefore, this paper proposes an angle-measurement scheme that corrects the angle-discrimination curve through SNR.

### 4.2. SNR-Adaptive Angle-Measurement Scheme

After obtaining the target sum-difference channel signals, the sum-channel SNR is calculated. Based on the SNR parameter and the pre-measured ideal angle-discrimination curve, an SNR-corrected angle-discrimination curve is generated. Subsequently, the sum-difference channel ratio result is substituted into the angle-discrimination curve to solve for the target angle. Two fundamental approaches exist:

Approach 1:

Generate the angle-discrimination curve under the current SNR condition. In this curve, the sum-channel SNR is identical for all wave arrival angles. The target angle is solved according to the angle-discrimination curve.

Approach 2:

Simulate the angle-discrimination curve from anechoic chamber testing, where the sum-channel SNR differs for all positions. Since noise is independent of wave arrival angle and can be regarded as a constant value, the SNR curve and the ideal sum-channel signal curve have a fixed ratio *k*.

The specific steps for Approach 2 are as follows:

(a) Substitute the ratio into the ideal angle-discrimination curve to obtain the target angle $\theta_{0}$;

(b) Based on the SNR and the sum-channel value corresponding to target angle $\theta_{0}$, solve for the fixed value *k*;

(c) Based on *k*, the ideal sum-channel signal curve, and the SNR curve, solve for the angle-discrimination curve;

(d) Correct the angle-discrimination curve using Equation (8) based on the SNR curve and ideal angle-discrimination curve;

(e) Substitute the ratio into the corrected angle-discrimination curve to obtain the target angle $\theta_{1}$;

(f) Repeat steps (b) to (e) until the solution satisfies the convergence condition $\Delta <0.001^{\circ }$.

**Figure 13:**
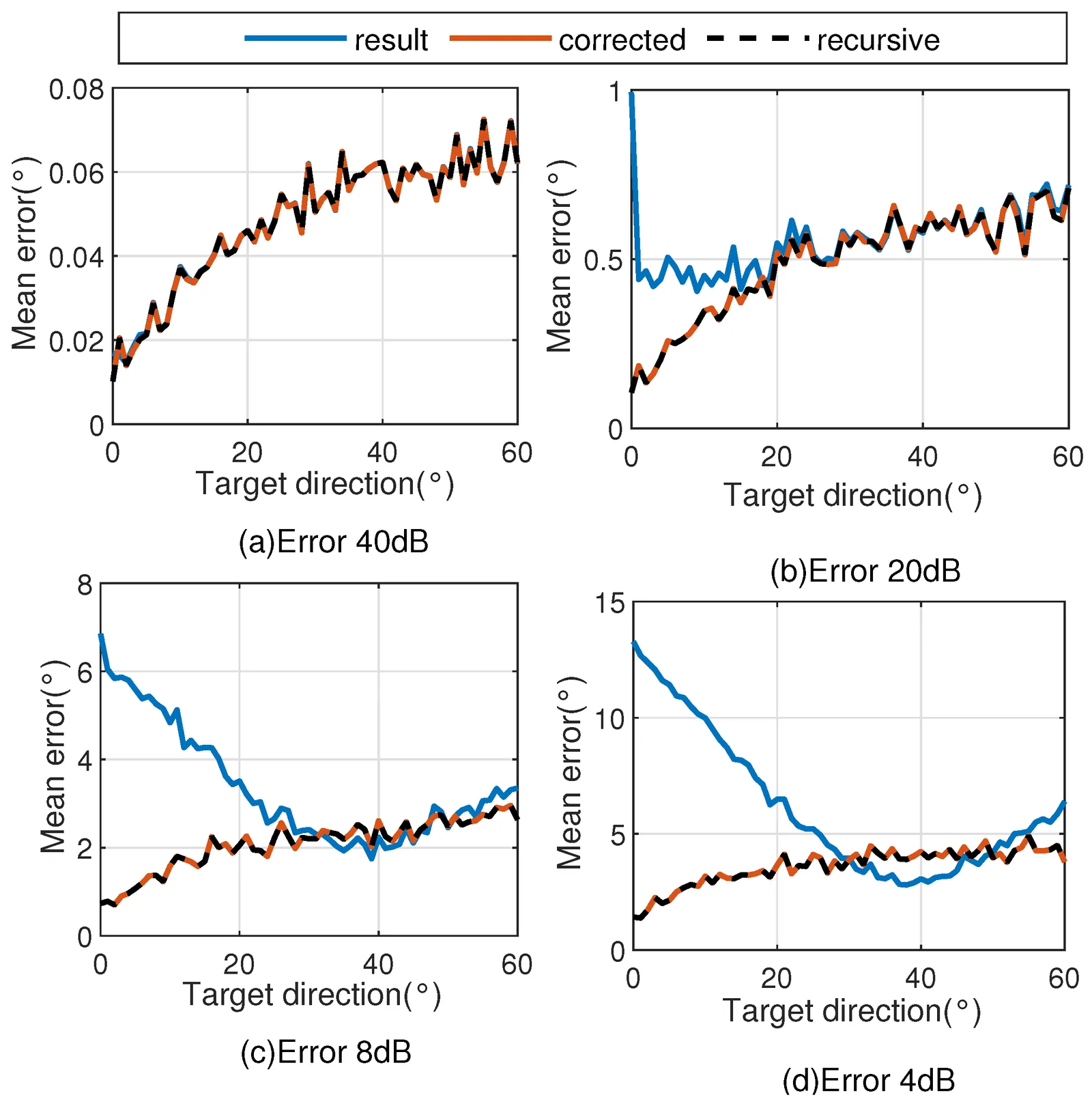
Average angle-measurement errors: (**a**) 40 dB; (**b**) 20 dB; (**c**) 8 dB; (**d**) 4 dB.

Figure 13 shows the average angle-measurement errors from 100 simulations, where (a), (b), (c), and (d) correspond to SNRs of 40 dB, 20 dB, 8 dB, and 4 dB, respectively. Blue represents the angle-measurement results using the ideal angle-discrimination curve directly; orange and black dashed lines represent the angle-measurement results from the two correction schemes.

From Figure 13, it can be observed that under high-SNR conditions, the angle-measurement error using the ideal angle-discrimination curve directly is very small, and the improvement from correction schemes is limited.

As the SNR decreases, the angle-measurement error using the ideal angle-discrimination curve directly gradually increases; as shown in Table 5, the angle-measurement errors at very small and very large wave arrival angles deteriorate severely. As the SNR decreases, the correction schemes significantly improve angle-measurement errors, and the error curves of the two correction schemes are essentially consistent, mutually verifying their effectiveness.

## 5. Warning System Test Results

In June 2025, Nanhu Laboratory completed antenna pattern testing of the warning system at the Microwave Anechoic Chamber Experimental Platform of the Yangtze Delta Research Institute (Huzhou), University of Electronic Science and Technology of China.

**Figure 14:**
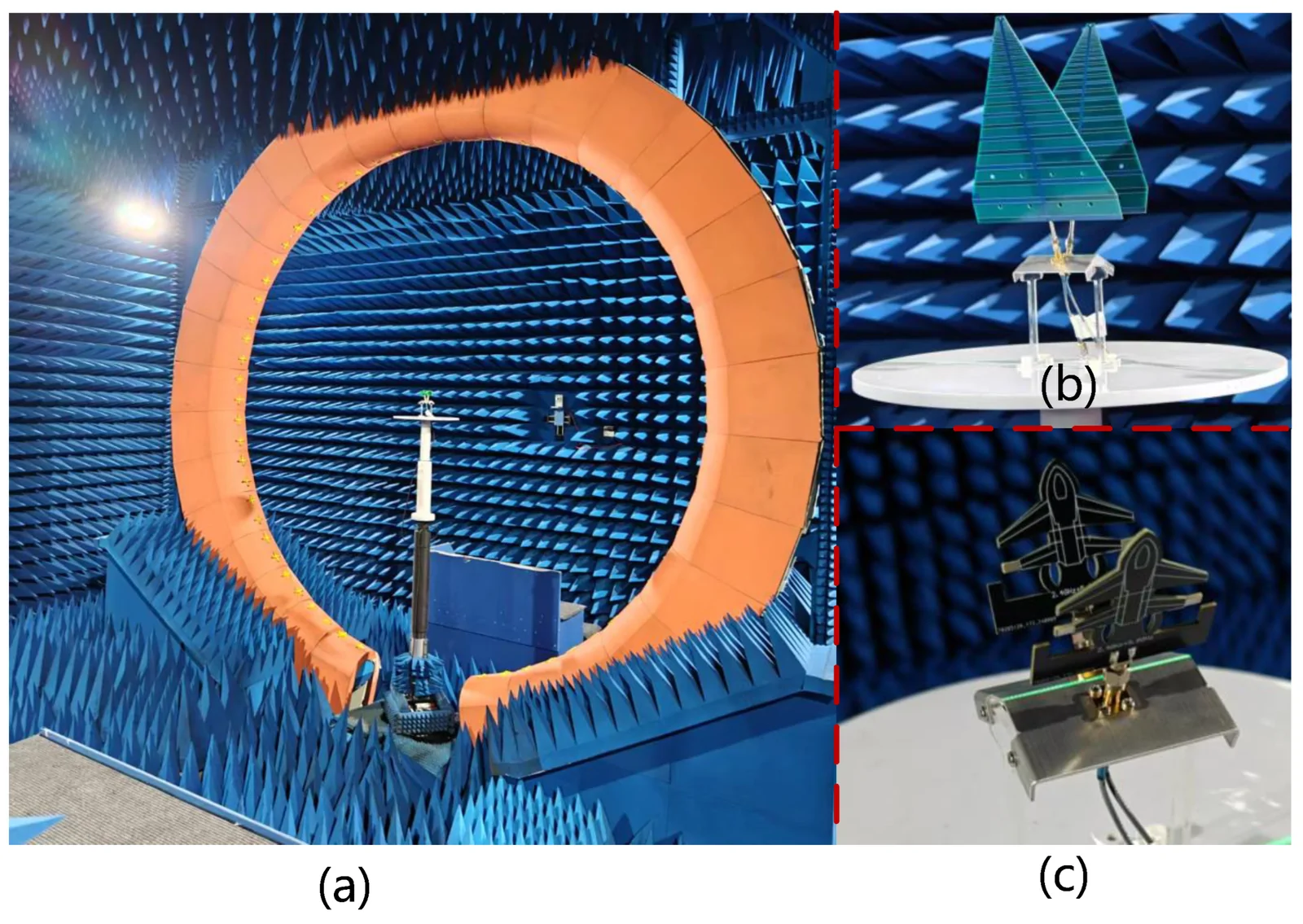
Anechoic chamber test environment: (**a**) Anechoic chamber; (**b**) antenna type 1; (**c**) antenna type 2.

Figure 14 shows the anechoic chamber test environment and schematic diagrams of two different receiving antennas mounted on the warning system. The anechoic chamber test results for Antenna 2 are presented below.

**Figure 15:**
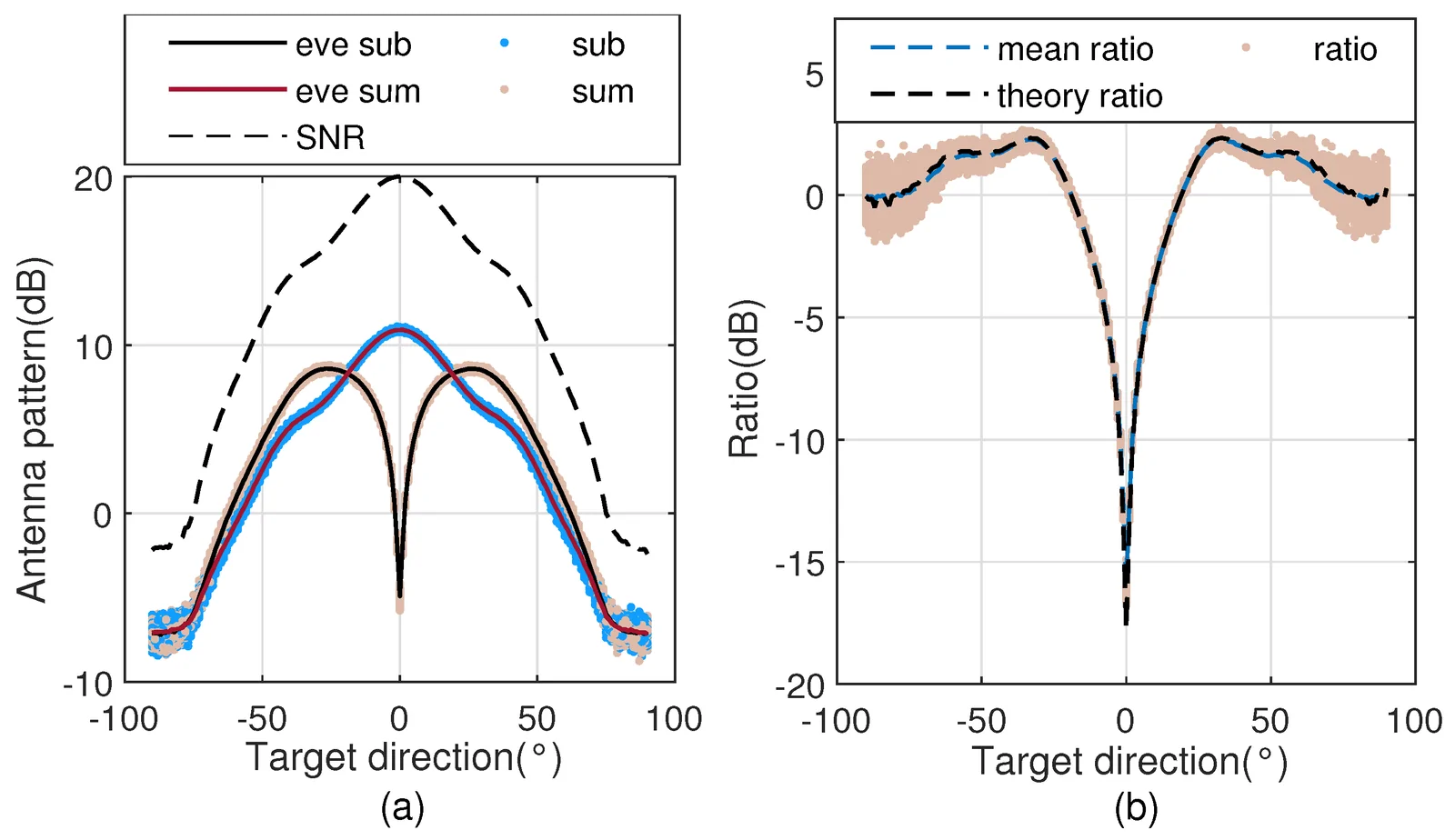
Test results: (**a**) Sum/difference channel Pattern and SNR curve, $0^{\circ }$ SNR =20 dB; (**b**) Angle detection curve, $0^{\circ }$ SNR =20 dB.

Figure 15 presents the anechoic chamber test results, corresponding to a sum-channel SNR of 20 dB at the boresight. In Figure 15a, blue scatter points represent the variation of the sum channel with target azimuth; orange scatter points represent the difference channel; red solid lines represent the average of 100 independent sum-channel results; black solid lines represent the difference channel average; black dashed lines represent the variation of sum-channel SNR with target azimuth.

In Figure 15b, orange scatter points represent the ratio data of the difference channel to the sum channel, and blue dashed lines represent the corresponding average. Based on the test results and SNR data, the angle-discrimination curve under ideal SNR conditions is calculated, corresponding to the black dashed line in the figure.

From Figure 15, it is evident that at the SNR of 20 dB, the angle-discrimination curve is essentially consistent with the ideal angle-discrimination curve. The SNR in anechoic chamber testing can be further improved, allowing test results to substitute for the ideal angle-discrimination curve.

**Figure 16:**
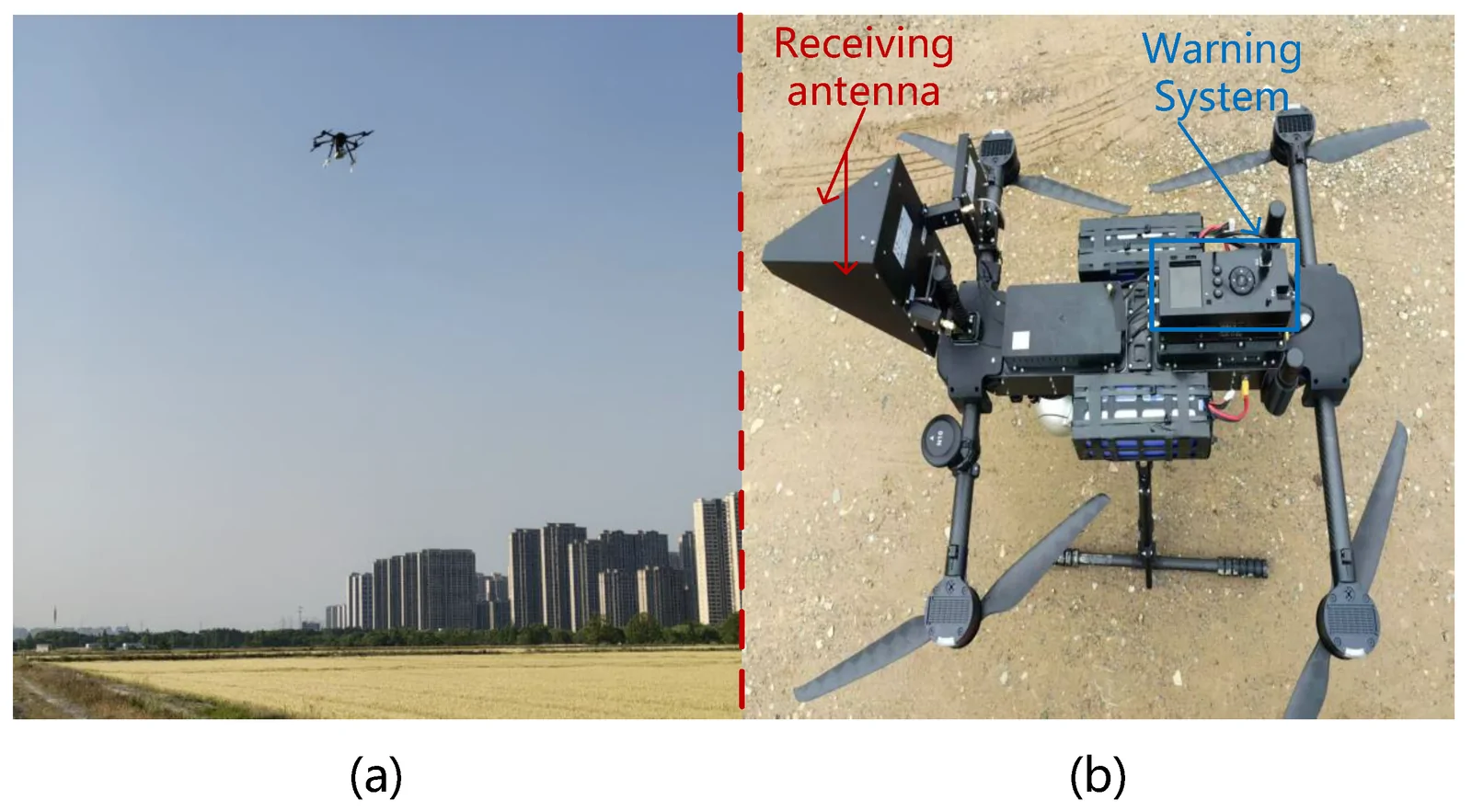
Field test environment and payload installation position: (**a**) Experimental environment; (**b**) airborne warning system.

**Figure 17:**
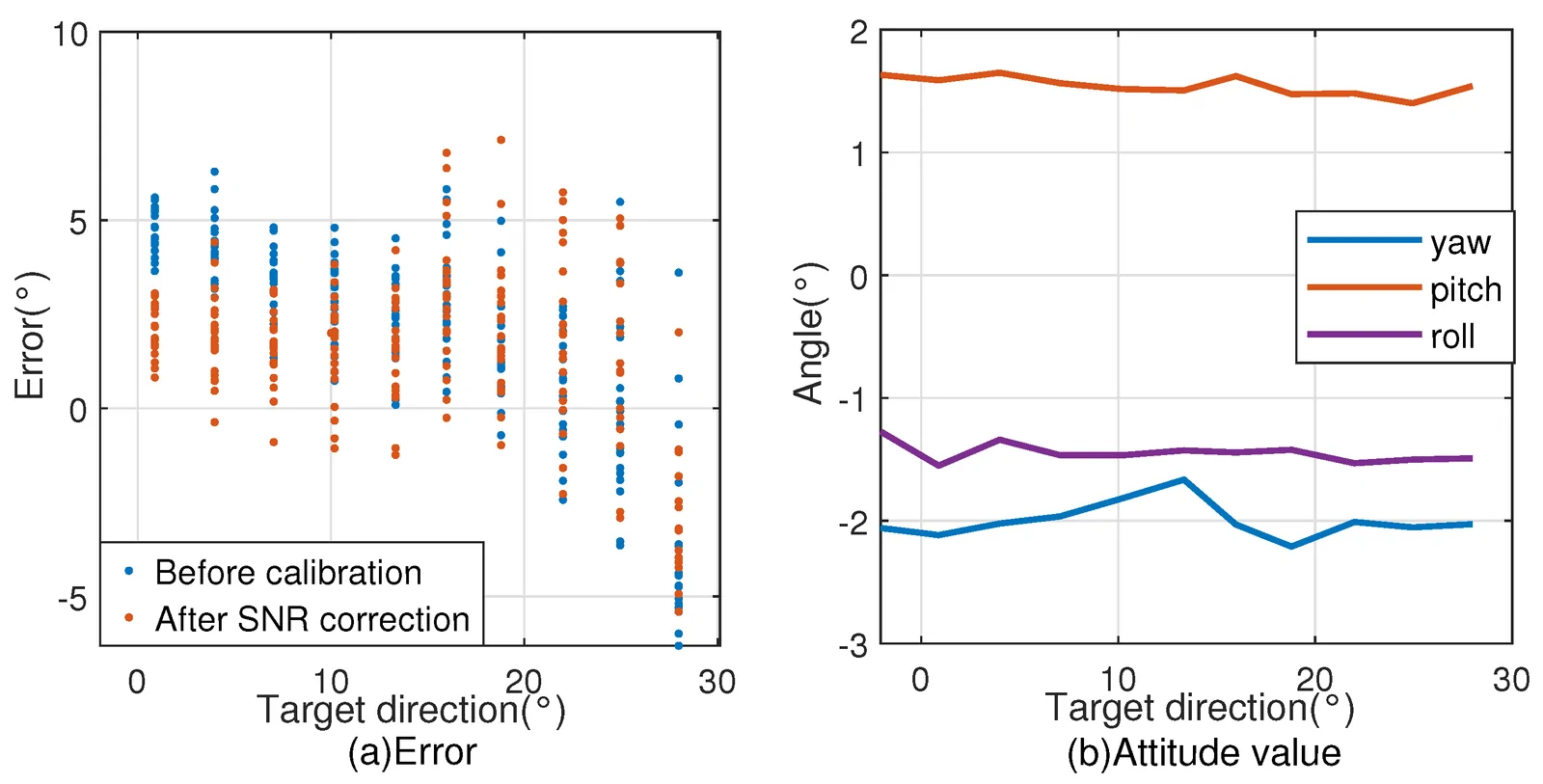
Angle-measurement errors and corresponding UAV attitudes: (**a**) angle-measurement errors; (**b**) UAV attitude data.

Figure 16 shows the field test environment and payload installation position schematic. The project team conducted field experiments in Shijiazhuang, Hebei Province, in May 2026. During the experiments, the warning system and target UAV were separated by 400 m, with a sum-channel SNR of 8 dB in the boresight direction. Eleven test points were selected in the approximately $0^{\circ }$ to $30^{\circ }$ range, with 20 independent test data obtained at each point. The results are shown in Figure 17.

Figure 17a presents the angle-measurement error results. The horizontal axis represents the true target azimuth, and the vertical axis represents the error between the measured result and the true target. Blue dashed lines represent the solution errors using the ideal (high-SNR) angle-discrimination curve; orange scatter points represent the solution errors using the corrected angle-discrimination curve.

Figure 17b shows the UAV attitude data corresponding to the test results, where blue, orange, and purple represent the yaw, pitch, and roll angles at different target azimuths, respectively. Select the most stable state of drone flight attitude for experimentation. Throughout the process, the average values of the three attitudes were approximately $\left[-2^{\circ },1.5^{\circ },-1.5^{\circ }\right]$.

**Figure 18:**
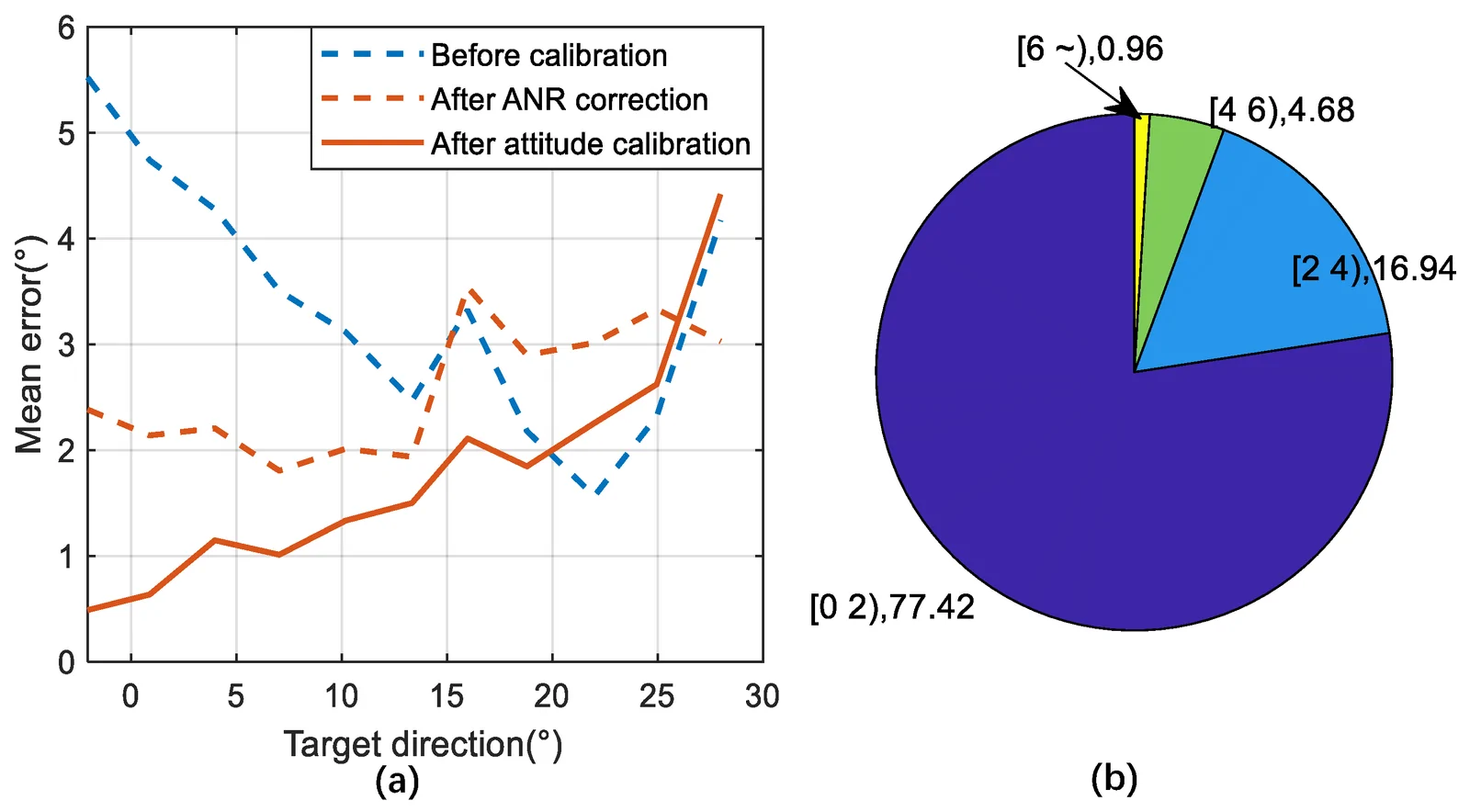
Attitude calibration results: (**a**) angle-measurement error comparison; (**b**) statistical distribution of calibrated errors.

Table 6 contains seven columns: Column 1 is the actual target azimuth; Columns 2∼4 are the UAV yaw, pitch, and roll angles, respectively; Column 5 is the angle-measurement error using the ideal angle-discrimination curve; Column 6 is the angle-measurement error using the SNR-corrected angle-discrimination curve. Based on the SNR-corrected angle-measurement results, the final calibration results using the three attitude data are presented in Column 7. These three columns of angle-measurement error data are shown in Figure 18.

In Figure 18a, the blue dashed line, orange dashed line, and orange solid line represent the angle-measurement errors using the ideal angle-discrimination curve, the SNR-corrected angle-discrimination curve, and the further attitude-calibrated results, respectively.

As shown by the blue and orange dashed lines in Figure 18a, the SNR-corrected angle-measurement error curve is significantly lower than the original results, with particularly pronounced improvement in the small-angle region. This validates the effectiveness of the real-time SNR correction scheme proposed in this paper.

As shown by the orange dashed line and solid line in Figure 18a, attitude calibration effectively improves angle-measurement accuracy across the full angular range. In extremely rare regions, the angle-measurement accuracy after attitude calibration shows slight degradation, which is attributed to the superposition of noise effects and attitude errors in large-angle regions with poor SNR. When the error signs are opposite and their effects cancel out, an illusion may occur where the uncalibrated data in certain regions exhibit higher accuracy. The statistical results of error values after attitude calibration are shown in Figure 18b, where over 77% of regions achieve angle-measurement accuracy better than $2^{\circ }$, fully validating the effectiveness of the attitude calibration method proposed in this paper.

## 6. Conclusions

This paper addresses the problem of degraded direction-finding accuracy in airborne warning systems under low-SNR and platform attitude perturbation conditions by proposing a direction-finding method that integrates real-time SNR correction with attitude adaptive calibration. Based on the monopulse amplitude–phase comparison angle-measurement principle, the proposed method estimates the received signal SNR online to dynamically correct the angle-discrimination curve while simultaneously utilizing attitude information from the onboard inertial measurement unit to establish coordinate system transformation relationships, thereby eliminating the influence of platform attitude on direction-finding results.

Simulation analysis demonstrates that using the ideal angle-discrimination curve directly under low-SNR conditions leads to significant angle-measurement errors, particularly in small-angle and large-angle regions. The proposed SNR-adaptive correction scheme effectively suppresses angle-measurement deviations caused by low SNR, and the two correction methods (direct generation of corrected curves and iterative convergence methods) exhibit equivalent error improvement effects. Anechoic chamber testing validates the consistency between the corrected angle-discrimination curve and the ideal curve under high-SNR conditions, providing a basis for using anechoic chamber test results as benchmarks.

Field experiments further validate the effectiveness of the proposed method in actual working environments. The results indicate that SNR correction significantly improves angle-measurement errors in the small-angle region; on this basis, attitude calibration improves direction-finding accuracy across the full angular range, with angle-measurement errors less than $2^{\circ }$ in over 77% of the test points. This result confirms the practical value of attitude calibration in airborne direction-finding applications.

This research provides a feasible technical path for the engineering implementation of airborne UAV warning systems. The residual measurement error of the system is mainly caused by IMU precision errors, SNR estimation deviations, and antenna pattern calibration errors. Future work will focus on error quantitative analysis and suppression, multi-target direction-finding algorithm optimization, robustness enhancement in complex electromagnetic environments, and multi-sensor information fusion to further improve system performance.

## Figures and Tables

**Table 1:** Basic design specifications of the warning system.

Parameter	Specification
Operating frequency range	800 MHz∼6 GHz
Instantaneous bandwidth	⩾150 MHz
Receiver sensitivity	⩽−105 dBm (1 MHz)
Detection range	⩾800 m
Multi-target detection	⩾5
Equivalent isotropic radiated power	⩾2 W @ 2.4 GHz
Detection time	⩽2 s
UAV identification	DJI, Autel, and other mainstream models
Warning mode	Audio
Volume	⩽465 mm × 135 mm × 50 mm
Power consumption	⩽40 W
Weight	⩽1 kg (excluding battery)

**Table 2:** Warning system configuration list.

Component	Specification
Antennas	1 set (3 units: 2 receive, 1 transmit)
RF front-end module	1
Baseband processing module	1
Display screen	1
Enclosure	1 set

**Table 3:** Attitude angle definitions.

Attitude	Angle Symbol	Rotation Axis	Positive Direction
Yaw	$\theta_{yaw}$	*z*	*x*-axis to *y*-axis
Pitch	$\theta_{pitch}$	*y*	*z*-axis to *x*-axis
Roll	$\theta_{roll}$	*x*	*y*-axis to *z*-axis

**Table 4:** Simulation configuration parameters.

Frequency	Antenna Angles	SNR	Sampling	Snapshots	Times
6 GHz	$10^{\circ }/-10^{\circ }$	40 dB/20 dB/8 dB/4 dB	60 GHz	100	100

**Table 5:** Comparison of angle-measurement errors before and after correction.

Angle	40 dB	20 dB	8 dB	4 dB
$0^{\circ }$	$0.003^{\circ }$	$0.186^{\circ }$	$2.250^{\circ }$	$4.430^{\circ }$
$2^{\circ }$	$0.008^{\circ }$	$0.151^{\circ }$	$1.741^{\circ }$	$3.547^{\circ }$
$4^{\circ }$	$0.011^{\circ }$	$0.150^{\circ }$	$1.401^{\circ }$	$2.921^{\circ }$
$6^{\circ }$	$0.014^{\circ }$	$0.168^{\circ }$	$1.094^{\circ }$	$2.078^{\circ }$
$8^{\circ }$	$0.014^{\circ }$	$0.161^{\circ }$	$0.909^{\circ }$	$1.451^{\circ }$
$10^{\circ }$	$0.016^{\circ }$	$0.145^{\circ }$	$0.603^{\circ }$	$0.989^{\circ }$
$12^{\circ }$	$0.016^{\circ }$	$0.145^{\circ }$	$0.488^{\circ }$	$0.817^{\circ }$
$14^{\circ }$	$0.014^{\circ }$	$0.143^{\circ }$	$0.483^{\circ }$	$0.856^{\circ }$
$16^{\circ }$	$0.013^{\circ }$	$0.110^{\circ }$	$0.630^{\circ }$	$1.153^{\circ }$
$18^{\circ }$	$0.011^{\circ }$	$0.107^{\circ }$	$0.627^{\circ }$	$1.277^{\circ }$
$20^{\circ }$	$0.007^{\circ }$	$0.090^{\circ }$	$0.616^{\circ }$	$1.117^{\circ }$
$22^{\circ }$	$0.004^{\circ }$	$0.063^{\circ }$	$0.424^{\circ }$	$0.910^{\circ }$
$24^{\circ }$	$0.001^{\circ }$	$0.028^{\circ }$	$0.227^{\circ }$	$0.440^{\circ }$

**Table 6:** Attitude data and error statistics.

Angle	Yaw	Pitch	Roll	Before Calibration	After SNR Correction	After Attitude Calibration
$-2.06^{\circ }$	$-2.06^{\circ }$	$1.63^{\circ }$	$-1.27^{\circ }$	$5.52^{\circ }$	$2.38^{\circ }$	$0.49^{\circ }$
$0.88^{\circ }$	$-2.12^{\circ }$	$1.59^{\circ }$	$-1.55^{\circ }$	$4.74^{\circ }$	$2.14^{\circ }$	$0.64^{\circ }$
$3.98^{\circ }$	$-2.02^{\circ }$	$1.65^{\circ }$	$-1.34^{\circ }$	$4.28^{\circ }$	$2.21^{\circ }$	$1.15^{\circ }$
$7.04^{\circ }$	$-1.97^{\circ }$	$1.56^{\circ }$	$-1.47^{\circ }$	$3.51^{\circ }$	$1.81^{\circ }$	$1.01^{\circ }$
$10.18^{\circ }$	$-1.82^{\circ }$	$1.52^{\circ }$	$-1.47^{\circ }$	$3.12^{\circ }$	$2.01^{\circ }$	$1.33^{\circ }$
$13.34^{\circ }$	$-1.67^{\circ }$	$1.50^{\circ }$	$-1.43^{\circ }$	$2.47^{\circ }$	$1.94^{\circ }$	$1.50^{\circ }$
$15.97^{\circ }$	$-2.03^{\circ }$	$1.62^{\circ }$	$-1.44^{\circ }$	$3.32^{\circ }$	$3.54^{\circ }$	$2.11^{\circ }$
$18.79^{\circ }$	$-2.21^{\circ }$	$1.47^{\circ }$	$-1.42^{\circ }$	$2.18^{\circ }$	$2.90^{\circ }$	$1.85^{\circ }$
$22.00^{\circ }$	$-2.01^{\circ }$	$1.48^{\circ }$	$-1.53^{\circ }$	$1.57^{\circ }$	$3.01^{\circ }$	$2.26^{\circ }$
$24.95^{\circ }$	$-2.05^{\circ }$	$1.40^{\circ }$	$-1.50^{\circ }$	$2.32^{\circ }$	$3.33^{\circ }$	$2.62^{\circ }$
$27.98^{\circ }$	$-2.03^{\circ }$	$1.54^{\circ }$	$-1.49^{\circ }$	$4.17^{\circ }$	$3.03^{\circ }$	$4.42^{\circ }$

## Data Availability

The original contributions presented in this study are included in the article. Further inquiries can be directed to the corresponding author.
